# Supplementary material for: Non–IgE- or Mixed IgE/Non–IgE-Mediated Gastrointestinal Food Allergies in the First Years of Life: Old and New Tools for Diagnosis
Source: Nutrients. 2021 Jan 14;13(1):226. doi: 10.3390/nu13010226 (PMC7829867; doi:10.3390/nu13010226)
Supplement: Supplementary file 1 [file nutrients-13-00226-s001.pdf]

Table S1. Search strategies and results in PubMed (MedLine) and EMBASE

Search Terms	Results on Pubmed.gov	Results on Embase.com	Non-overlapping Records
"Non IgE mediated food allergy" AND "infant"	178	292	405
"Non IgE mediated food hypersensitivity" AND "infant"	156	282	380
"Eosinophil esophagitis" AND "infant"	221	87	298
"Eosinophilic gastroenteritis" AND "Infant"	42	53	81
"Eosinophilic colitis" and "infant"	47	26	61
"Food Protein Induced Enteropathy" and "Infant"	240	436	594
"Food Protein Induced Proctocolitis"	60	74	108
"Food Protein Induced Enterocolitis"	352	300	578
"non IgE mediated food allergy" AND "patch test"	111	64	173
"Non IgE mediated food allergy" AND "infant" AND "endoscopy"	2	16	17
"Non IgE mediated food hypersensitivity" AND "infant" AND "endoscopy"	2	15	16
"Eosinophil esophagitis" AND "infant" AND "endoscopy"	118	57	160
"Eosinophilic gastroenteritis" AND "infant" AND "endoscopy"	17	20	35
"Food Protein Induced Enteropathy" AND "infant" AND "endoscopy"	7	32	33
"Food Protein Induced Proctocolitis" AND "endoscopy"	5	38	38
"Food Protein Induced Enterocolitis" AND "endoscopy"	6	37	37
"CoMiSS" AND "food allergy"	11	22	29
"CoMiSS" AND "cow's milk allergy"	14	23	33
"CoMiSS" AND "cow's milk protein allergy"	11	20	28
"CoMiSS" AND "non IgE mediated food allergy"	2	3	3
"Cow's milk related score" AND "food allergy"	33	32	49
"Cow's milk related score" AND "cow's milk allergy"	38	43	65
"Cow's milk related score" AND "cow's milk protein allergy"	25	31	46
"Cow's milk related score" AND "non IgE mediated food allergy"	5	4	6
"Symptom based score" AND "food allergy"	150	83	194
"Symptom based score" AND "cow's milk allergy"	37	35	57
"Symptom based score" AND "cow's milk protein allergy"	31	25	43
"Symptom based score" AND "non IgE mediated food allergy"	6	7	10
"Faecal biomarkers" AND "cow's milk allergy"	7	5	11
"Faecal biomarkers" AND "non IgE mediated food allergy"	7	4	10
"Faecal biomarkers" AND "food allergy"	22	22	40
"Faecal calprotectin" AND "cow's milk allergy"	11	30	33
"Faecal calprotectin" AND "non IgE mediated food allergy"	8	9	16

"Faecal calprotectin" AND "food allergy"	23	61	75
"Faecal IgA" AND "cow's milk allergy"	2	11	11
"Faecal IgA" AND "non IgE mediated food allergy"	1	2	2
"Faecal IgA" AND "food allergy"	19	4	13
"Faecal tumor necrosis factor" AND "cow's milk allergy"	1	8	8
Faecal tumor necrosis factor AND non IgE mediated food allergy	2	5	6
Faecal tumor necrosis factor AND food allergy	5	18	22
Faecal eosinophil cationic protein and cow's milk allergy	2	1	2
Faecal eosinophil cationic protein AND non IgE mediated food allergy	1	0	0
Faecal eosinophil cationic protein AND food allergy	5	3	5
β-defensin AND cow's milk allergy	1	7	7
βeta-defensin AND non IgE mediated food allergy	0	3	3
β-defensin AND food allergy	3	69	71
α-1 antitripsin AND cow's milk allergy	2	14	14
α-1 antitripsin AND non IgE mediated food allergy	1	4	4
α-1 antitripsin AND food allergy	5	55	56
Eosinophil derived neurotoxin AND cow's milk allergy	3	6	8
Eosinophil derived neurotoxin AND non IgE mediated food allergy	3	8	9
Eosinophil derived neurotoxin AND food allergy	12	34	40
Faecal occult blood AND cow's milk allergy	8	27	34
Faecal occult blood AND non IgE mediated food allergy	2	9	10
Faecal occult blood AND food allergy	7		50
IgG AND IgG4 AND non IgE mediated food allergy	15	21	33
IgG AND IgG4 AND non IgE mediated gastrointestinal food allergy	3	7	9
IgG AND IgG4 and "cow milk allergy"	12	10	33
"Laboratory findings" AND non IgE gastrointestinal food allergies	2	3	3
IgG AND IgG4 AND Eosinophilic esophagitis	29	23	49
IgG AND IgG4 AND Food Protein Induced Enterocolitis	2	5	5
"Lymphocyte stimulation test" AND non IgE mediated food allergy	4	23	2
"Lymphocyte stimulation test" AND "cow milk allergy"	2	15	15
"Laboratory findings" AND non IgE gastrointestinal food allergies	2	10	11
"Lymphocyte stimulation test" AND Eosinophilic esophagitis	0	1	1
"Lymphocyte stimulation test" AND Food Protein Induced Enterocolitis	6	18	19
TOTAL	2287	2864	4318
